# Temporal trends of a cellular host response test for sepsis and a comparison with selected biomarkers of inflammation and infection

**DOI:** 10.1038/s41598-025-14860-w

**Published:** 2025-08-20

**Authors:** Matthew S. Berlinger, Matt Sorrells, Christopher B. Thomas, Tonya Jagneaux, James E. Walker, Jennifer Daigle, Heather Quiriarte, Youyoung Kim, Guillaume Spielmann, Debbie Robertson, Susan Park Ochsner, Gideon Avornu, Katherine Fenstermacher, Elijah James Kurien, Camille King, Adenine Cembellin-Kao, Chase Yonamine, Querino Maia, Maciej Obrebski, David N. Hager, Roya Sheybani, Henry T. K. Tse, Ajay M. Shah, Robert Scoggins, Hollis R. O’Neal, Richard Rothman

**Affiliations:** 1 Our Lady of the Lake Regional Medical Center, 5246 Brittany Drive, Baton Rouge, LA 70808 USA; 2 Cytovale, Inc, San Francisco, CA USA; 3 Franciscan Missionaries of Our Lady Health System, Baton Rouge, LA USA; 4 Louisiana State University Health Sciences Center, New Orleans, LA USA; 5 Louisiana State University, Baton Rouge, LA USA; 6 Johns Hopkins University, Baltimore, MD USA; 7 Kootenai Health, Coeur d’Alene, ID USA; 8 Department of Emergency Medicine, Johns Hopkins University, Baltimore, MD USA

**Keywords:** IntelliSep Index (ISI), Sepsis diagnosis, Immune activation, Sepsis biomarkers, Organ dysfunction, Antimicrobial stewardship, Infectious diseases, Bacterial infection, Infection, Infectious diseases

## Abstract

Sepsis, a condition characterized by immune dysregulation, is the leading cause of in-hospital mortality and requires rapid treatment. Assessing immune dysregulation has been challenging. The IntelliSep Index (ISI), a novel biomarker which leverages microfluidic deformability cytometry to assess immune activation, has been evaluated as a test for Emergency Department use but it is unclear how the signal evolves as patient condition evolves. In a 47-patient cohort with hospital stays ≤ 14 days, ISI provided a good indicator of disease progression by blinded physician review. ISI trends correlated with clinical improvement over time. During the first 12 h following presentation, ISI was unaffected by antibiotic treatment initiated in the emergency department (ED). After this period however, ISI values declined, reflecting the patient’s response to treatment, with Band 3 patients showing a 20% decline within the first two days of admission. ISI trends preceded Sequential Organ Failure Assessment (SOFA) score changes by two days. ISI also provided more stable and timely disease state assessments compared to traditional biomarkers (PCT, CRP, IL-6, and neutrophil elastase). Larger studies are needed to validate these findings and assess ISI’s clinical utility as a prognosticator of sepsis progression and treatment response.

**Supplementary Information:**

The online version contains supplementary material available at 10.1038/s41598-025-14860-w.

## Introduction

Sepsis is a prevalent and costly condition in hospitals, and early recognition and treatment is critical to the prevention of adverse outcomes^1^. Though sepsis has been defined as a syndrome characterized by “life-threatening organ dysfunction caused by a dysregulated host response to infection”, a means to assess the dysregulated host response has been lacking^2,3^. The assessment of immune dysregulation is critical to the diagnosis and management of patients during the course of their treatment.

The IntelliSep test (Cytovale, San Francisco, USA), was recently developed with the explicit intended use of facilitating rapid assessment of immune dysregulation and sepsis risk^4^. This FDA-cleared, in-vitro diagnostic, assesses cellular host response via deformability cytometry of leukocyte biophysical properties where changes in cell shape under fluidic stress serve as an indicator of immune activation. Interpretation is based on the sample’s IntelliSep Index (ISI), an ordinal measurement that ranges between 0.1 and 10.0 and is further divided into three “bands” representing the probability of sepsis being present as previously described^5^. These bands represent increasing risk of sepsis from Band 1 (low probability) to Band 3 (high probability sepsis). Previous work has established the diagnostic and prognostic performance of this test in patients with suspected infection presenting to the Emergency Department (ED)^4,6^. However, it is not known if temporal changes in ISI correlate with clinical course, response to treatment, or both.

In this study we characterize (1) how ISI changes following initiation of treatment for sepsis, (2) how ISI correlates with patient illness severity over time (as measured by the Sequential Organ Failure Assessment (SOFA) score and blinded retrospective physician assessment), and (3) how the performance of ISI changes as compared to other biomarkers (PCT, CRP, IL-6, and neutrophil elastase) in a prospectively recruited population of ED patients presenting with suspected sepsis.

## Methods

### Study population and characteristics

#### Enrollment criteria

##### Inclusion criteria

Study performance sites included Our Lady of the Lake Regional Medical Center (OLOL) in Baton Rouge, Louisiana, and Johns Hopkins University Hospital (JHU) in Baltimore, MD. Subjects eligible for this study were adults 18 years of age or older who provided informed consent and exhibited two or more Systemic Inflammatory Response Syndrome (SIRS) criteria, at least one of which was an abnormal temperature or white blood cell (WBC) count. In an effort to enrich the population for patients who ultimately were more likely to develop sepsis, participation also required blood culture collection, antibiotic administration, and hospital admission. Lastly, to assess disease resolution over a 7-day measurement period (see below), only subjects with a length of stay (LOS) of 14 days or less were included in the analysis. These criteria ensured that the study population was appropriately defined, and that the data collected were relevant to the objectives of the research. Relatively few subjects had a LOS greater than 14 days, complicating the analysis of this group. For completeness, these cases are presented in the Supplemental Information.

##### Exclusion criteria

Patients were excluded if they were expected to transition to comfort measures during the ED visit or were receiving an investigational medication prior to blood collection. Based on the IntelliSep 510(k) clearance (K220991) labelling requirements, patients with any of the following were also excluded: received a cytotoxic chemotherapeutic agent or other excluded medication within the past three months; had a known history of hematological malignancy (leukemia, lymphoma, myeloma, myelodysplastic syndrome, or other myeloproliferative disorder); or had undergone a hematopoietic stem cell transplant or any solid organ transplant. In addition, Participants were excluded if there were too few timepoints (defined as less than 2 days of measurements); the initial sample was not tested within the IntelliSep test’s 5-h stability window; or the first measurement was > 24 h post ED admission. Lastly, patients unable to participate in study-related activity and those with blood collections not occurring within the requirements of the protocol were excluded.

### Blood collection by study site

For participants at OLOL, blood sample collection was initiated within 12 h of the first recorded vital sign in the ED, with additional measurements taken over a period of up to 7 days during the hospital course. At JHU, blood sample collection began within 24 h of the first recorded vital sign in the ED, with subsequent samples collected over the same 7-day period. To account for the differing enrollment settings at each site, time zero was standardized to the triage time (defined as the time of the first recorded vital sign). At OLOL, participants were required to be admitted to the hospital, while at JHU, due to higher ED boarding times, participants were either admitted or had an admission order placed during the ED encounter. At both sites, subsequent EDTA blood samples were collected during the hospital course.

### Time series analysis

A time series analysis was conducted to elucidate the temporal relationship between ISI and disease progression in patients undergoing treatment. An initial blood sample was drawn upon presentation during the patient’s initial ED visit and ISI was recorded upon patient consent. Subsequently, two blood samples per day were collected for the first two days, with the second sample collected 12 ± 6 h after the initial sample. These samples were intended to allow analysis of ISI changes in response to initial clinical interventions delivered in the ED and shortly upon admission. Enrolled patients were grouped by initial ISI band score, and band changes from these groups were recorded at 12 h and 24 h. Beyond the first two days, blood samples were collected once daily for days 3–7 or until discharge, whichever came first. The analysis included patients with a LOS of less than or equal to 14 days.

Due to the variability in time sampling intervals for ISI testing among subjects, ISI values were plotted and then interpolated to obtain seven equally distributed timepoints, representing days 0 to 7 in one-day increments. ISI levels were then analyzed across different initial interpretation bands on the day of presentation (first measurement), on day 2 (for those that had a length of stay of two or more days), and at the final measurement.

### Clinical condition and SOFA score analysis

To further assess the relationship between ISI trends and clinical outcomes, aggregate trends from the time series analysis were compared with the clinically-assessed disease state (sepsis, infection but not sepsis, or no infection), course and clinical acuity, as determined through retrospective physician adjudication. Adjudication was performed by an expert site-associated physician, who was blinded to the ISI results and independently reviewed the medical records of all enrolled patients.

For each day a patient was enrolled in the study, the adjudicator assessed their clinical condition as ‘improved’, ‘remained stable’ or ‘worsened’, up to a maximum of seven days. These trends were quantified by assigning a value of 10 at the beginning of the patient’s visit and adjusting by − 1 or + 1 daily based on whether the patient improved or worsened, respectively.

The comparison of ISI trends, clinical condition, and clinical acuity as measured by SOFA scores was conducted by analyzing time series data for each patient. Total and per organ system SOFA scores were aggregated for each patient at baseline and daily (using the worst value during the calendar day) for up to 7 days or discharge, whichever came first. Baseline values were subtracted from subsequent measurements to track changes over time. SOFA scores were also calculated daily and aggregated across patients for comparison. This analysis allowed for an assessment of ISI trends in relation to organ dysfunction resolution, providing insights into the utility of ISI as an early indicator of clinical improvement.

### Biobanking and biomarker analysis

In addition to participation in the measurement of their ISI over time, participants were also asked whether or not to consent to having their samples biobanked for further biomarker analysis. The decision did not influence the ability of the patient to participate in this study. These plasma samples were collected and frozen at – 80 °C. The biomarkers measured in this study included neutrophil elastase, PCT, CRP, and IL-6.

All enzyme-linked immunosorbent assays (ELISAs) were performed according to the manufacturers’ protocols. Neutrophil elastase levels were measured using the Human Neutrophil Elastase ELISA Kit (AB270204; Abcam, Cambridge, MA, USA). PCT levels were assessed with the Human Procalcitonin ELISA Kit (EHPCT; Thermo Fisher, Waltham, MA, USA). CRP concentrations were quantified using the Human C-Reactive Protein/CRP ELISA Kit (DCRP00B; Quantikine, Minneapolis, MN, USA). IL-6 levels were measured with the Human IL-6 HS ELISA (HS600C; Quantikine, Minneapolis, MN, USA).

To assess the performance of the ISI relative to traditional biomarkers, aggregate trends from the time series analysis were compared between patients adjudicated as infected and non-infected. Correlations between ISI and traditional biomarkers (CRP, IL-6, neutrophil elastase, and PCT) were established by analyzing the temporal relationships and trends of these markers in relation to the clinical course of disease resolution.

### Statistical analysis

As this was an exploratory study, no power calculation was performed to determine sample size. Trends and correlations between serial ISI measurements and disease course progression were assessed, along with clinical assessments and severity metrics.

When comparing aggregate ISI values across time points, we utilized one-way ANOVA with Tukey’s Honestly Significant Difference Test to compute statistical significance and *P*-values across groups. An alpha level of 5% was used for all analyses, unless otherwise stated. One-way ANOVA was selected for its ability to compare means across multiple groups, with Tukey’s test applied post-hoc to identify specific differences between groups. This approach was chosen due to the study’s focus on analyzing variations in ISI across different time points. The same approach was used in the analysis of other biomarkers.

Data analysis was performed using appropriate statistical methods and software. Descriptive statistics are presented as means, standard deviations, medians, and ranges for the continuous variables, and as counts and percentages for categorical variables. Demographics were tabulated overall and by relevant populations. Two-sided confidence intervals were provided where appropriate.

### Safety hazards identification

All safety hazards associated with the biomarker assays and handling of biological samples were identified and managed in accordance with institutional guidelines. Proper personal protective equipment (PPE) and laboratory safety protocols were strictly followed to ensure the safety of all personnel involved in the study.

### Ethical approval

Ethical approval for this study was obtained from the Institutional Review Boards (IRBs) at both participating institutions (WCG IRB: 20223071; JHU IRB: 00347448). The study was conducted in accordance with the principles of the Declaration of Helsinki. All participants provided informed consent prior to their inclusion in the study. For studies involving human samples, appropriate ethical approval and patient consent procedures were adhered to, ensuring the ethical and respectful treatment of all participants.

### Analytic framework

To achieve the goal of characterizing (1) how ISI changes following initiation of treatment for sepsis, (2) how ISI correlates with patient illness severity over time, and (3) how the performance of ISI changes as compared to PCT, CRP, IL-6, and neutrophil elastase. Time series data were analyzed for (1) changes in initial measurements over the first 12–24 h of monitoring, (2) distribution of sepsis status and aggregate ISI transitions over time, (3) a comparison of ISI trends with Clinical Condition and SOFA scores, and finally, (4) a comparison of ISI with traditional biomarkers.

## Results

### Study population and characteristics

Two sites enrolled a total of 68 patients. Supplementary Fig. S1 details selection of the study analysis population. OLOL enrolled 24 patients, with 2 excluded due to issues with the initial time point. JHU enrolled 44 patients. Seven were excluded for not meeting study enrollment criteria, and 4 were excluded due to a limited number of timepoints collected.

Although measurements were taken for a maximum of 7 days, 8 patients had a hospital length of stay of 15 days or more. Given that these patients had more days without measurements than with, they were not included in the analyses presented. Instead, clinical vignettes, retrospective daily assessments of clinical condition, and ISI values measured for the 7 timepoints, for these patients are presented in Supplementary Table S1.

In total, 47 patients were included in the final analysis. Among these patients, the adjudicated sepsis prevalence was 29.8% (N = 14). Baseline characteristics and comparative statistical descriptions for the patients per site are shown in Table 1. The initial ISI band distribution at OLOL was: 3 patients in Band 1, 9 patients in Band 2, and 8 patients in Band 3. The initial ISI band distribution at JHU was: 8 patients in Band 1, 10 patients in Band 2, and 9 patients in Band 3. Median time to the first ISI collection was 4.4 h (IQR 3.7–5.1) and 17.0 h (IQR 12.8–19.8) at OLOL and JHU, respectively.

**Table 1:** Demographics and baseline characteristics for patients per site.

Category	OLOL	JHU	*P*-value
	N = 20	N = 27	
Age, median (Q1–Q3)	62.5 (55.8–77.2)	40.0 (33.0–57.5)	*p* < 0.001
Biological sex, N (%)			
Female	9 (45.0)	16 (59.3)	ns
Male	11 (55.0)	11 (40.7)	ns
Race, N (%)			
Black	6 (30.0)	11 (40.7)	ns
White	11 (55.0)	12 (44.4)	ns
Other	3 (15.0)	4 (14.8)	ns
Lactate			
Measured, N (%)	18 (90.0)	21 (77.8)	ns
Median (Q1–Q3)	1.8 (1.4–2.4)	2.2 (1.8–2.8)	ns
Blood culture			
Collected N (%)	20 (100.0)	26 (96.3)	ns
Positive N (%)	9 (45.0)	3 (11.1)	*p* < 0.01
SOFA day of enrollment, median	2.0	1.0	*p* < 0.01
Requirement for mechanical ventilation, N (%)	1 (5.0)	0 (0.0)	ns
Sepsis, by retrospective physician adjudication, N (%)	8 (40.0)	6 (22.2)	ns
Admitted to ICU, N (%)	3 (15.0)	3 (11.1)	ns
Hospital length of stay, median (Q1–Q3)	5.0 (3.0–7.2)	8.0 (6.0–9.5)	*p* < 0.05
28-Day mortality, all-cause N (%)	1 (5.0)	0 (0.0)	ns
IntelliSep Index, median (Q1–Q3)	4.3 (3.1–4.6)	3.9 (3.3–5.2)	ns

Biomarker measurements were performed on plasma samples of 19 patients at OLOL and 11 patients at JHU that had provided consent for the remnant of their samples to be banked for further biomarker analysis. These samples had the following distribution across the ISI Bands: OLOL—Band 1: 3 patients, Band 2: 9 patients, Band 3: 7 patients; JHU—Band 1: 3 patients, Band 2: 5 patients, Band 3: 3 patients.

### Time series analysis

#### Early ISI measurements within 12 and 24 h

To assess the ISI response early in the stay, the stability of the ISI was assessed at three time points: upon presentation (Day 0), approximately 12 ± 6 h later, and approximately 24 h later (Day 1). Within the first 12 h, no significant changes were observed among patients initially classified in Band 1 or Band 3. Specifically, all patients whose ISI value was in Band 1 at presentation remained in Band 1 at the second measurement (taken 12 ± 6 h after the first sample), and all patients initially classified in Band 3 remained in Band 3 over the same time period.

**Fig. 1:**
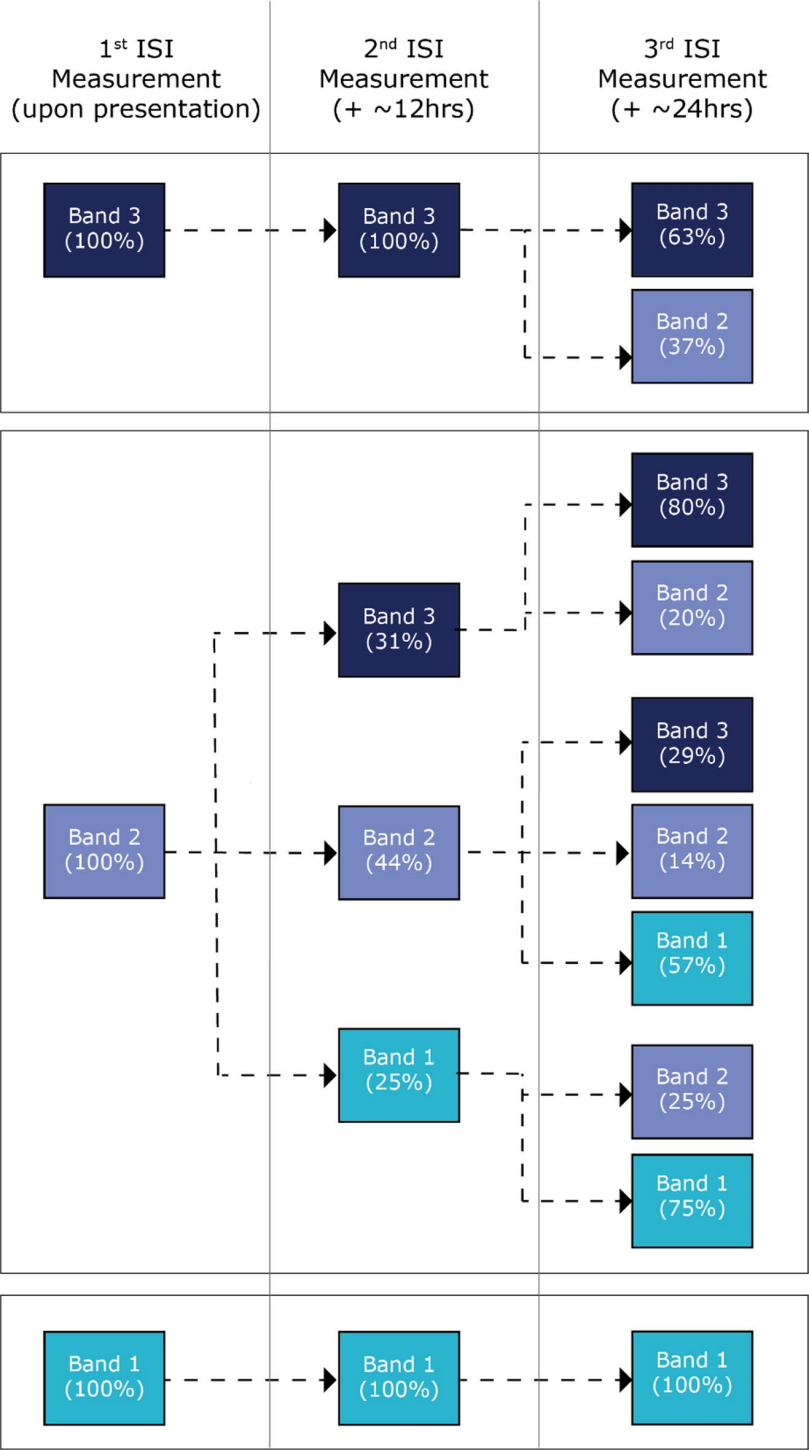
Time series analysis: early ISI measurements (within 12 and 24 h). This figure shows ISI measurement transitions across three time points: upon presentation, approximately 12 h later, and approximately 24 h later. The data illustrate that patients in Bands 1 and 3 generally remain stable within the same band over the first 12 h, whereas those in Band 2 show greater variability, with transitions to either Band 1 or Band 3 by the third measurement.

In contrast, patients with an initial ISI value in Band 2 exhibited greater variability in measurement across time. By the 12-h mark, 54% of these patients had transitioned to either Band 1 or 3. In total, for this first time period, approximately one-third of Band 2 patients transitioned to Band 3, a quarter to Band 1 and the rest remained in Band 2. By the 24-h mark, 94% of all Band 2 patients had transitioned to either Band 1 or Band 3. The majority of patients who started in Band 2 and transitioned to Band 1 or 3 at the first interval time point (12 h) remained in that same band by the 24-h (Day 1) mark (Fig. 1).

#### Distribution of sepsis status and aggregate ISI transitions across bands over time

**Fig. 2:**
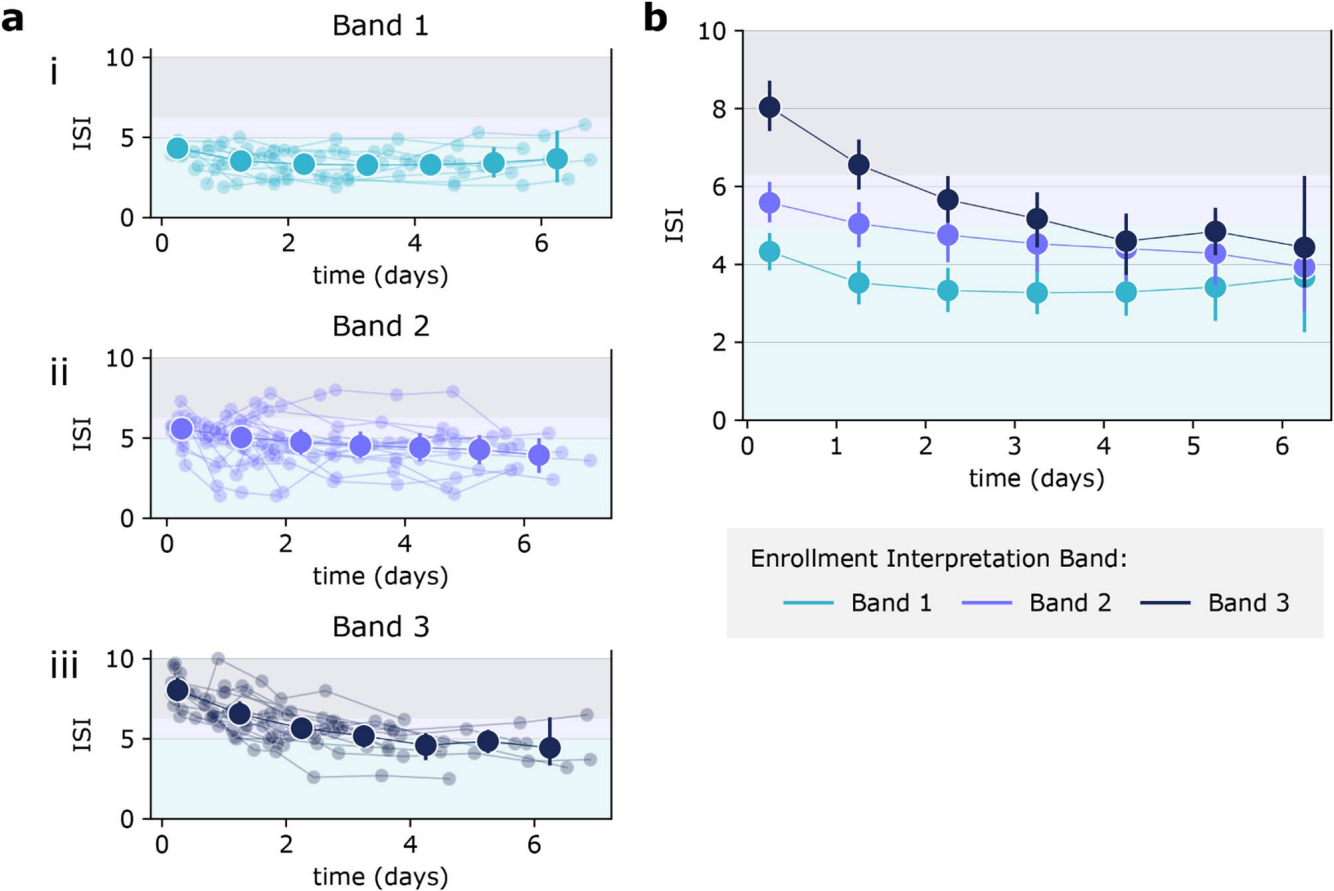
Observed changes in ISI levels over time. (**a**) Overlay of ISI trends for all patients, with aggregate trends shown for (i) Band 1, (ii) Band 2, and (iii) Band 3. Error bars represent standard error. (**b**) Aggregate trends of ISI over time across initial interpretation bands for patients included in the time series analysis. Error bars represent standard error.

The initial distribution of patients by ISI interpretation band and sepsis status is shown in Table 2, with 9% of Band 1 patients adjudicated to have sepsis, compared to 10% in Band 2 and 69% in Band 3. Figure 2 illustrates the observed changes in ISI over time, including the progression and transitions of ISI across bands. While each patient’s trajectory is unique, in order to assess the overall trend in sequential ISI measurements, timepoints for patients grouped by their initial ISI band are aggregated and interpolated into one trendline per Band as shown in Fig. 2a, subplots i–iii.

**Table 2:** Interpretation band and sepsis status (as determined by retrospective physician adjudication) of patients included in the time series analysis.

Initial IntelliSep band	Sepsis status		
	Negative	Indeterminate	Positive
Band 1 (n = 11)	9	1	1
Band 2 (n = 20)	16	2	2
Band 3 (n = 16)	4	1	11

*Initial Band 1*

For patients initially classified in Band 1 (N = 11), a minor but statistically insignificant decrease in ISI was observed over time (Fig. 2b).

*Initial Band 2*

For patients initially classified in Band 2 (N = 20), despite different clinical trajectories in the first 24 h, overall there was a significant drop in ISI, transitioning from Band 2 (mean ISI 5.6) to Band 1 (mean ISI 4.8) with a near-constant decrease observed from Day 0 to Day 2 (for those with a length of stay of two or more days) (*P* < 0.05). A further significant decrease was noted from Day 0 to the final measurement (mean ISI 3.9, *P* < 0.0001) (Fig. 2c).

*Initial Band 3*

For patients initially classified in Band 3 (N = 16), there was on average a decrease in ISI from Day 0 to Day 2. This decrease resulted in patients moving from Band 3 (mean ISI 8.0), to Band 2 (mean ISI 5.7) (Fig. 2b). As the patients’ hospital stay continued, there was on average a slower but consistent decrease in ISI from days 2 to 6. For this time period ISI moved from Band 2 (mean ISI 5.7) to Band 1 (mean ISI 4.4).

Notably, the rate of change in ISI for initial Band 3 patients during days 2–6 (when they reached ‘mid-Band 2’ and continued decreasing to Band 1), was similar to that of initial Band 2 patients during days 0–4, when they too dropped from ‘mid Band 2’ to Band 1 (Fig. 2b). Also of note, are the significant reductions in ISI observed for Band 3 patients from Day 0 to Day 2 (*P* < 0.05), and from Day 0 to their final measurement on Day 7 or Day of Discharge, whichever came first (*P* < 0.0001) (Fig. 2c).

### Comparison of ISI trends with clinical condition and SOFA scores

**Fig. 3:**
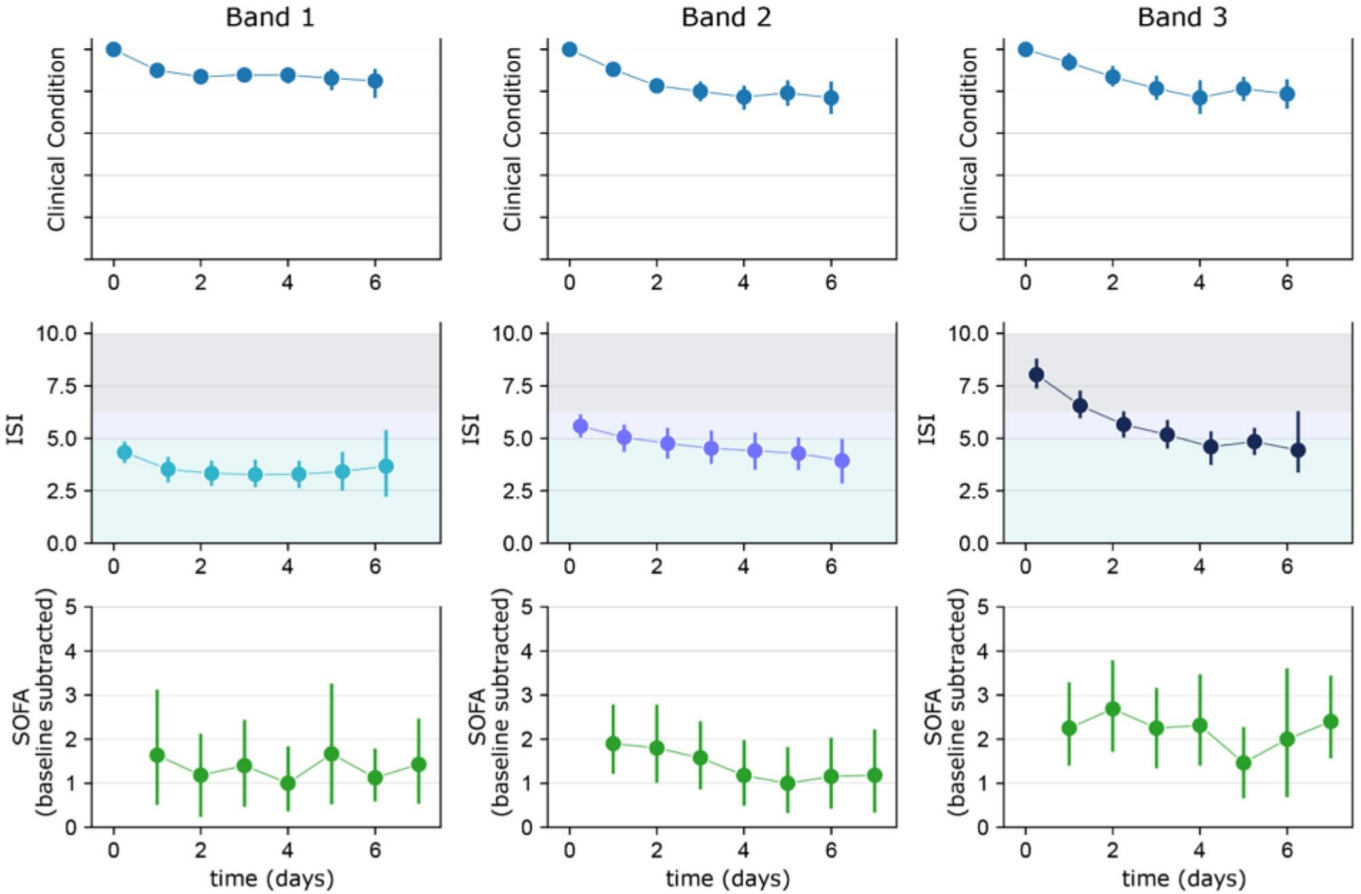
Comparison of ISI trends with clinical condition and SOFA scores. Trends in ISI, an assessment of clinical condition retrospectively provided by a physician with access to the medical record, and total SOFA score over time by initial interpretation Band are shown. Clinical condition was assessed daily using a scoring system in which the score was adjusted by − 1 or + 1 based on whether the patient improved or worsened, respectively. Error bars represent standard error.

When comparing ISI and SOFA score changes over time, significant decreases in ISI were observed between time points, while no significant changes were observable for SOFA scores (Fig. 3). Of note, decreasing ISI and clinical improvements observed in Band 2 and Band 3 patients were not consistently reflected in the SOFA scores, even at the per-system level suggesting that the SOFA score may be a lagging indicator of clinical improvement. Renal and respiratory systems were the two most important organ systems contributing to the total SOFA score, with both systems having a similar magnitude of effect (Supplementary Fig. S2).

For patients whose ISI at presentation was in Band 2 or 3, the ISI was observed to closely reflect the evolution of daily clinical assessments. Specifically, ISI appeared to be a leading indicator of organ dysfunction resolution. On the other hand, serial SOFA scores did not show a significant correlation to clinical condition or ISI. For example, patients initially classified in Band 2 had a decrease in ISI values from Day 0 to Day 1, while trends in SOFA scores lagged, showing a decrease from days 2 to 4. In Band 3, patients exhibited a rapid downward trend in the ISI from the onset of measurement, which correlated with a swift improvement in clinical condition. Trends with SOFA scores in these patients showed no discernible pattern of change.

### Comparison of ISI with traditional biomarkers

Aggregate trends from the time series analysis were compared between infected and non-infected patients to assess the performance of the ISI relative to traditional sepsis biomarkers.

**Fig. 4:**
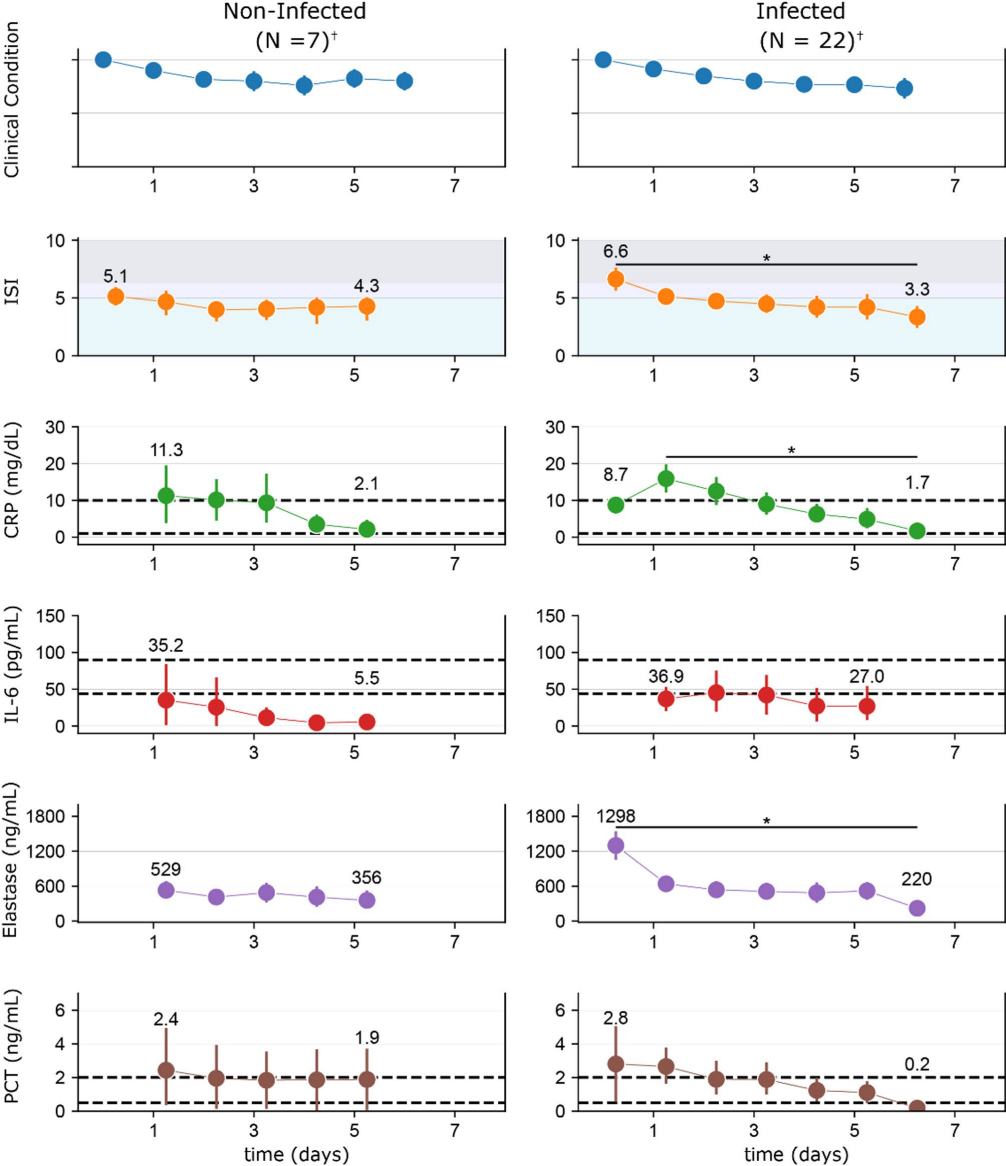
Comparison of ISI with traditional biomarkers. Biomarker trends across time among infected and non-infected patients for ISI, CRP, IL-6, neutrophil elastase, and PCT, (* indicates *P* < 0.05) †: N = 1 patient with indeterminate infection status was within this cohort. Unfilled time points among biomarkers are due to missing data. For each biomarker, dashed lines represent commonly used cutoffs of severity/disease risk.

In the infected subpopulation, ISI demonstrated a stronger reflection of the clinical course of disease resolution than traditional biomarkers such as PCT, CRP, and IL-6 (Fig. 4). IL-6 and PCT biomarkers showed delayed responses relative to observed disease resolution. CRP levels increased initially and then decreased among infected patients. In contrast, ISI aligned with clinical assessments and corresponded to observed changes in condition throughout the patient’s course. Trends for these biomarkers between septic and non-septic patients and correlations between the ISI and the other biomarkers tested are provided in the Supplementary Information (Supplementary Figs. S3 and S4, respectively).

## Discussion

This study demonstrates the kinetics of the ISI over time among patients who present to the ED with suspected sepsis. These results have potential implications in the interpretation of the ISI as a diagnostic marker after treatment begins to monitor the response to treatment, and, along with future research, to help evaluate the usefulness of the marker in the prediction of sepsis resolution and recovery.

IntelliSep results, by interpretation band, remain largely stable over the first 24 h of care, with most patients outside of the transitory Band 2, having the same result at 12 and 24 h that they had during the initial measurement. Most importantly, no patient with a low-risk, Band 1 result at 24 h had an initial high-risk, Band 3 result. This finding addresses a key limitation in current cultures-based sepsis diagnostics and supports the potential of IntelliSep test to provide diagnostic value in the first 24 h of a patient’s journey without the concern for interference from active interventions like antibiotic administration and resuscitation.

After this initial period of stability, the resolution curve for patients initially classified in Band 3 generally reaches Band 2 within two days. Subsequently, all patients initially classified in Bands 2 or 3 follow a similar pattern of resolution, independent of their initial interpretation band. For the entire population, the ISI decreased on average over time and was observed to be reflective of the clinical condition as assessed by an external physician adjudicator. Furthermore, the trajectory of ISI appears to provide a signal that more closely approximates clinical condition than SOFA scores, which had limited correlation in this study and typically improve slowly as organ function normalizes.

The lack of SOFA score correlation to clinical condition and ISI trends is not unexpected. The ISI is an assessment of the state of immune activation, and because the organ dysfunction of sepsis is felt to be immune-mediated, the anticipated finding is that resolution of immune activity leads to resolution of organ dysfunction. Furthermore, many components of the SOFA (Neuro, Cardiovascular, and Respiratory) are somewhat dependent on clinician behavior (for example, with the use of sedatives, vasoactive agents, and ventilatory support) and clinicians typically discontinue these interventions upon clinical resolution of organ dysfunction.

All enrolled patients had clinical evidence of systemic inflammation as defined by the inclusion criteria of having 2 or more SIRS criteria. Despite these inclusion criteria, approximately one quarter of enrolled patients (7/29) were not adjudicated as infected and a majority were not adjudicated to have sepsis, emphasizing the need to differentiate systemic inflammation from the immune dysregulation that underlies infection-related sepsis. Notably, the signal for both CRP and IL-6 appeared to be associated more closely with the clinical evolution of non-infected patients than it did with infected ones, suggesting the markers may have greater usefulness as a general marker of inflammation rather than a specific indicator of infectious processes.

For infected patients, inflammatory markers such as CRP and IL-6 exhibited broadly delayed responses relative to clinical improvement and ISI. PCT showed wide distribution of initial values as well as a slightly delayed resolution relative to clinical condition and ISI. These differences in performance both initially and upon resolution may indicate that ISI provides a more immediate indication of patient condition.

Neutrophil elastase is a serine protease stored in the primary granules of neutrophils and released upon neutrophil activation. It is associated with neutrophil extracellular trap (NET) release, which is a major factor in the deformability characteristics measured by the ISI^7^. Not surprisingly, initial neutrophil elastase levels correlates most strongly with initial ISI; however, these levels fall rapidly after initiation of therapy. This volatility, as well as its poorly defined threshold values, limits use of this biomarker to a research tool.

The alignment between ISI trends and clinical assessments suggests that the ISI has potential to provide real-time evaluation of patient condition, contrasting with measurements of organ dysfunction that may persist despite resolution of the underlying pathologic process that caused the organ dysfunction. Furthermore, by directly assessing immune activation, ISI may provide a clearer and earlier insight into disease progression compared to other biomarkers that result from inflammation or response to infection. The results of this analysis support further evaluation of ISI to more thoroughly understand its alignment with the biological mechanisms that underlie the clinical syndrome of sepsis over time.

If these associations are established, IntelliSep could enhance clinical care of sepsis patients by providing a stable metric of immune dysregulation that correlates closely with patient condition. This supports the idea that the test could improve clinical decision-making and patient management at multiple points during admission, enabling optimized care based on the evolution of patient condition, which may lead to improved outcomes and greater efficiency.

Though this study suggests that incorporating ISI into sepsis diagnostic protocols could enhance the ability to monitor disease progression and respond more effectively in initial stages of sepsis management, it is a small pilot observational study. Thus, the generalizability of the findings and their translation into actionable results is limited. While the analysis aimed to aggregate results to draw broader conclusions, the uniqueness of each patient’s clinical course introduces variability that may influence the observed trends and their applicability to a wider population.

Moreover, this pilot study focused on the majority of patients who experienced LOS under 15 days. The smaller number of patients with LOS 15 days or longer had more complex trajectories that typically worsened and, per protocol, had more days without measurements. This study, therefore, provides information primarily about how the ISI evolves in patients with an improving clinical trajectory, not in those with a deteriorating condition. Further studies with larger patient populations are required to adequately study ISI evolution for patients whose condition worsens.

The results of this study highlight the potential of the ISI as a tool to monitor a patient’s immune activation state. If confirmed by larger studies, these insights into immune activation could lead to more timely interventions and more efficient care. Despite the pilot study’s limitations, the evidence suggests that a cellular host response test has promise as a tool to augment clinical management of potential sepsis patients. Future research with larger, more diverse patient populations will be crucial in confirming these findings, examining ISI evolution in patients whose condition worsens, and further defining the role of ISI in the active management of patients with sepsis.

## Supplementary Information

Supplementary Information.

## Data Availability

The datasets generated and analyzed during the current study are available from the corresponding author on reasonable request. Due to the nature of the data, some restrictions may apply to the availability of patient information and clinical datasets, but access may be granted with appropriate approvals.
