# Supplementary material for: Temporal trends of a cellular host response test for sepsis and a comparison with selected biomarkers of inflammation and infection
Source: Sci Rep. 2025 Aug 20;15:30501. doi: 10.1038/s41598-025-14860-w (PMC12368155; doi:10.1038/s41598-025-14860-w)
Supplement: Supplementary file 1 — Supplementary Information. [file 41598_2025_14860_MOESM1_ESM.docx]

**Evaluating the Temporal Performance and Prognostic Efficacy of a Cellular Host Response Test for Sepsis: A Comparison with Conventional Biomarkers**

**Supplementary Information**


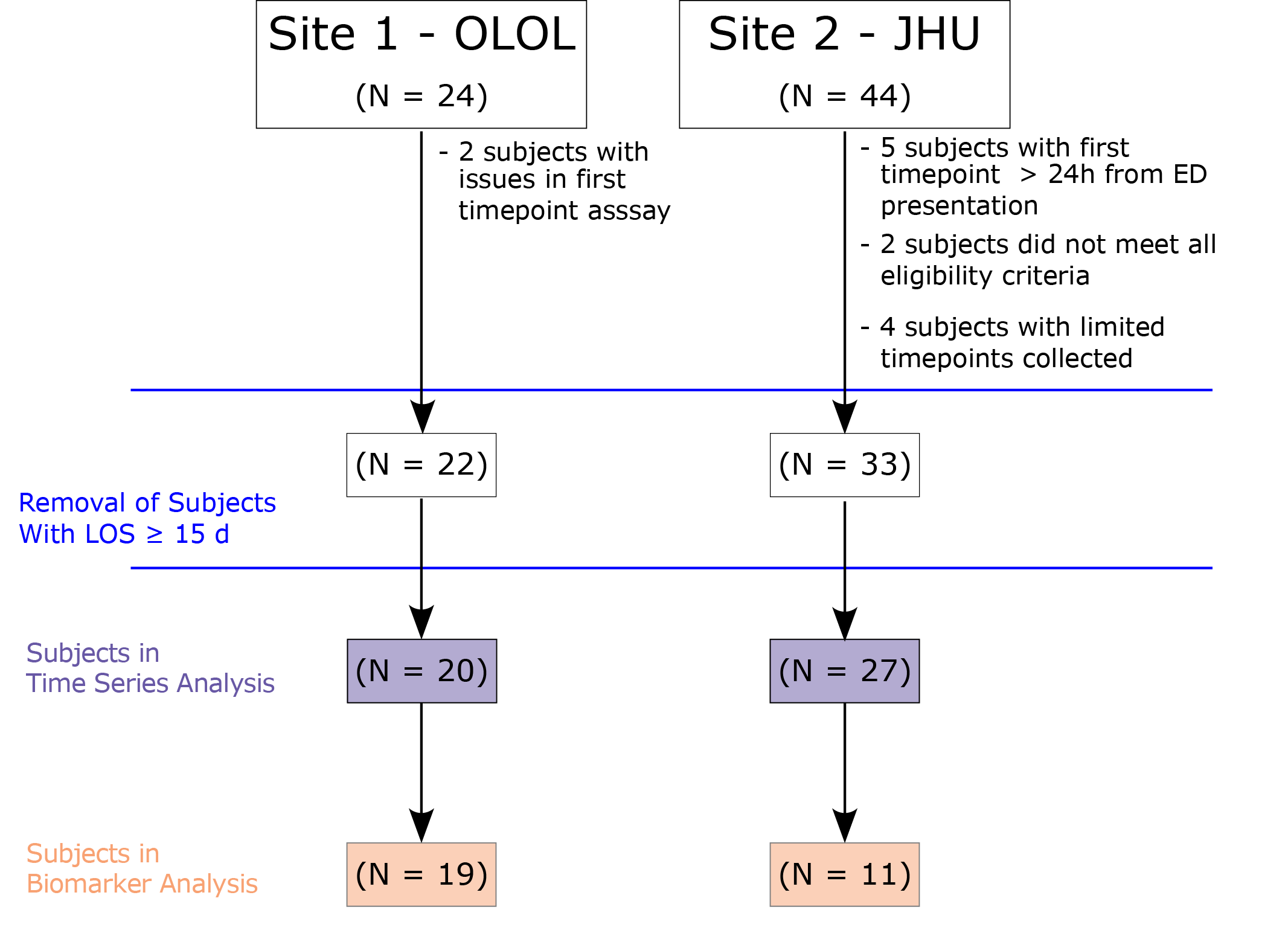


**Supplementary Figure S1.** Selection of final subject population for the study.

**Supplementary Table S1:** Clinical Vignettes and ISI Values for Patients with LOS ≥ 15 Days

| Site 1 – OLOL | Patient ID 001 | - 50-year-old male - History of CVA, DM, Dyslipidemia / Hyperlipidemia, HTN. - Lactate was not measured in the ED. - WBC on day of presentation to the ED = 18.8 ×10^3^/µL. - Received antibiotics (Piperacillin-Tazobactam) and fluids in the ED. - ED diagnosis: perineal abscess, history of diabetes mellitus, cellulitis of groin. - Admitted to floor/non-critical unit. - Blood cultures negative (x2); Abscess cultures (1) positive for Prevotella bivia, (2) negative. - Hospital length of stay = *18 days* (inclusive of day of ED presentation and day of discharge), then discharged to LTAC. - Discharge diagnosis: necrotizing soft tissue infection of perineum. - Retrospective adjudication: Infection of Skin/Skin Structure source; no organ dysfunction; **no sepsis**. | 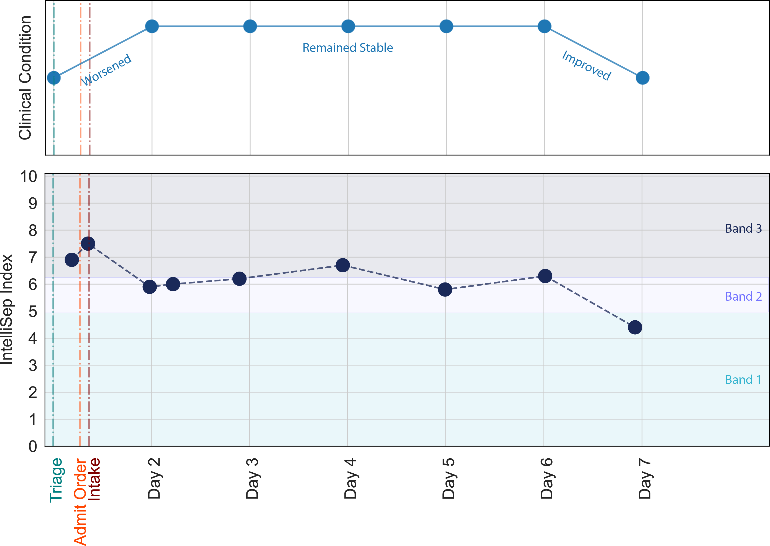   \|  \| **Day 1** \| **Day 2** \| **Day 3** \| **Day 4** \| **Day 5** \| **Day 6** \| **Day 7** \| \| --- \| --- \| --- \| --- \| --- \| --- \| --- \| --- \| \| SOFA \| 1 \| 2 \| 1 \| 1 \| 1 \| 0 \| 0 \| |
| --- | --- | --- | --- | --- | --- | --- | --- | --- | --- | --- | --- | --- | --- | --- | --- | --- | --- | --- | --- |
|  | Patient ID 027 | - 71-year-old male - History of Afib, CHF, Dyslipidemia / Hyperlipidemia, HTN, Intracranial hemorrhage. - Lactate measured in the ED = 0.9 mmol/L. - WBC on day of presentation to the ED = 24.8 ×10^3^/µL. - Received antibiotics (Aztreonam) and fluids in the ED. - ED diagnosis: confusion, urinary tract infection with hematuria (site unspecified), closed head injury (initial encounter), atrial fibrillation with RVR. - Admitted to floor/non-critical unit. - Blood cultures positive for Klebsiella spp. (x2). - Hospital length of stay = *17 days* (inclusive of day of ED presentation and day of discharge), then discharged to nursing home/SNF. - Discharge diagnosis: sepsis, thrombocytopenia, OSA (obstructive sleep apnea). - Retrospective adjudication: Infection of Urine & Urine System / Genitourinary source; CNS, CV, RESP, & RENAL organ dysfunction; **sepsis**. | 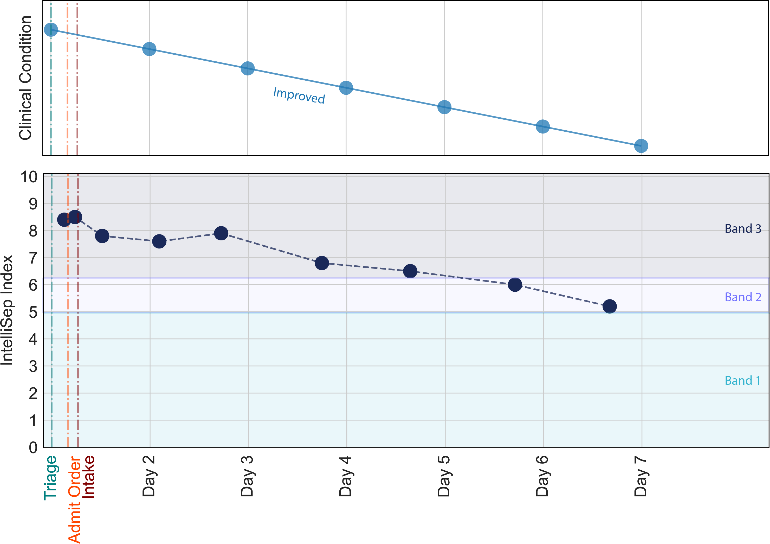   \|  \| **Day 1** \| **Day 2** \| **Day 3** \| **Day 4** \| **Day 5** \| **Day 6** \| **Day 7** \| \| --- \| --- \| --- \| --- \| --- \| --- \| --- \| --- \| \| SOFA \| 5 \| 3 \| 5 \| 5 \| 3 \| 3 \| 2 \| |
| Site 2 – JHU | Patient ID 005 | - 52-year-old male - History of DM. - Lactate measured in the ED = 1.6 mmol/L. - WBC on day of presentation to the ED = 16.6 ×10^3^/µL. - Received antibiotics (Ceftriaxone, Metronidazole, Piperacillin/Tazobactam) in the ED. - ED diagnosis: perforated appendicitis. - Admitted to floor/non-critical unit. - Blood cultures negative (x2); Urine culture negative. - Hospital length of stay = *25 days* (inclusive of day of ED presentation and day of discharge), then discharged home. - Discharge diagnosis: perforated appendicitis, leukocytosis, small bowl perforation. - Retrospective adjudication: Infection of Gastrointestinal / Abdominal source; CV, RENAL, & GI organ dysfunction; **sepsis**. | 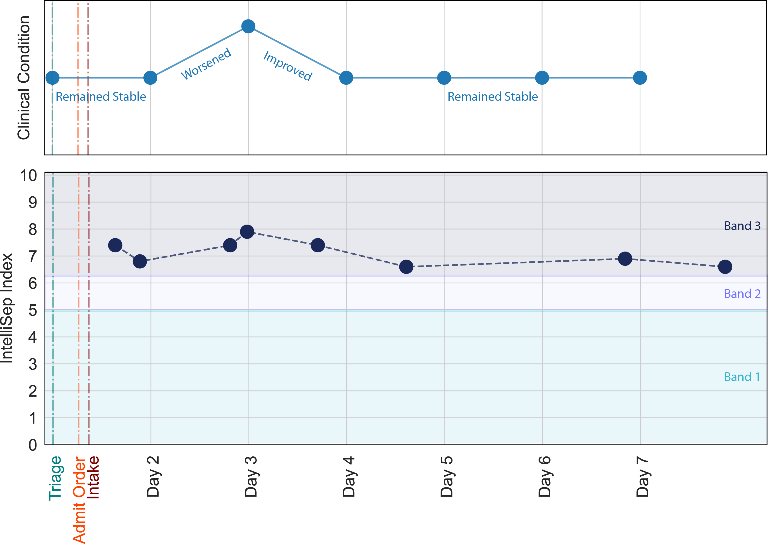**Sample for Day 5 was not drawn.*   \|  \| **Day 1** \| **Day 2** \| **Day 3** \| **Day 4** \| **Day 5** \| **Day 6** \| **Day 7** \| \| --- \| --- \| --- \| --- \| --- \| --- \| --- \| --- \| \| SOFA \| 0 \| 1 \| 3 \| 3 \| 2 \| 4 \| 4 \| |
|  | Patient ID 012 | - 56-year-old male - History of Anemia, GERD, HTN, Degenerative Disc Disease, Seizures, Sarcoidosis, Arthritis, Benign Prostatic Hyperplasia, Chronic Elevated CPK, Interstitial Lung Disease, Liver Disease. - Lactate measured in the ED = 2.4 mmol/L. - WBC on day of presentation to the ED = 14.6 ×10^3^/µL. - Received antibiotics (Cefepime) in the ED. - ED diagnosis: fever (unspecified cause), at risk for infectious disease due to recent foreign travel, bilateral lower extremity edema. - Admitted to floor/non-critical unit. - Blood cultures (1) positive for Salmonella spp., (2) negative, (3) negative; Joint fluid culture (1) positive for Salmonella spp., (2) negative; Bone tissue culture negative. - Hospital length of stay = *19 days* (inclusive of day of ED presentation and day of discharge), then discharged home. - Discharge diagnosis: right hip septic arthritis, salmonella arthritis, bacteremia, intramuscular fluid collection, fever due to angioedema. - Retrospective adjudication: Infection of CV and Bone & Joint source; no organ dysfunction; **no sepsis**. | 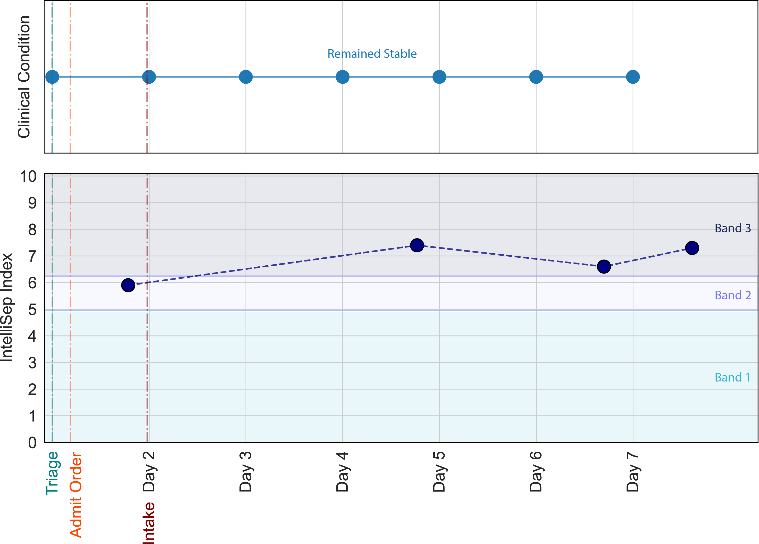 **Samples for Day 2, 3, & 5 were not drawn.*   \|  \| **Day 1** \| **Day 2** \| **Day 3** \| **Day 4** \| **Day 5** \| **Day 6** \| **Day 7** \| \| --- \| --- \| --- \| --- \| --- \| --- \| --- \| --- \| \| SOFA \| 1 \| 1 \| 1 \| 0 \| 0 \| 0 \| 0 \| |
|  | Patient ID 019 | - 48-year-old male - History of HIV, HTN. - Lactate measured in the ED = 1.5 mmol/L. - WBC on day of presentation to the ED = 40.0 ×10^3^/µL. - Received antibiotics (Azithromycin, Cefepime, Ceftriaxone) in the ED. - ED diagnosis: sepsis, acute respiratory infection. - Admitted to floor/non-critical unit*, elevated to ICU within 4 days*. - Blood culture negative (x1); Urine culture positive for Klebsiella oxytoca/Raoultella ornithinolytica; Sputum culture positive for yeast; Respiratory panel negative; SARS-CoV-2 negative. - Hospital length of stay = *31 days (13 ICU days)* (inclusive of day of ED presentation and day of discharge), then deceased. - Discharge diagnosis: N/A. - Retrospective adjudication: Infection of Respiratory source; RENAL organ dysfunction; **sepsis**. | 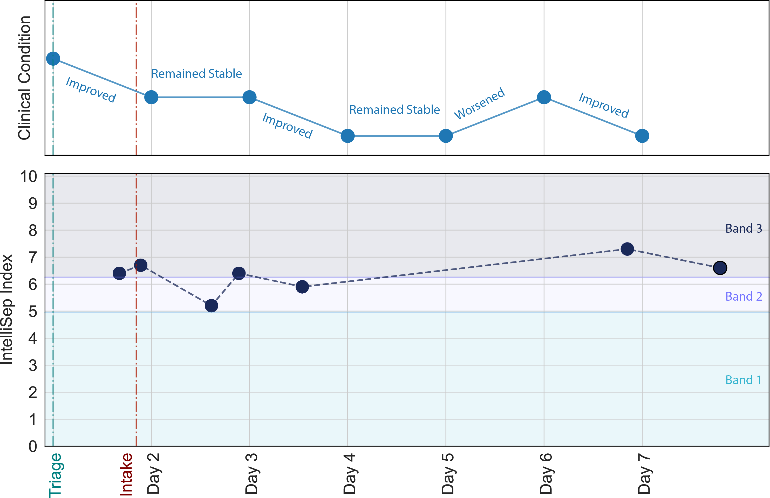**Samples for Day 4 & 5 were not drawn.*   \|  \| **Day 1** \| **Day 2** \| **Day 3** \| **Day 4** \| **Day 5** \| **Day 6** \| **Day 7** \| \| --- \| --- \| --- \| --- \| --- \| --- \| --- \| --- \| \| SOFA \| 2 \| 0 \| 3 \| 1 \| 2 \| 3 \| 3 \| |
|  | Patient ID 035 | - 71-year-old female - History of Anemia, Pleural Effusion, Colitis. - Lactate measured in the ED = 1.5 mmol/L. - WBC on day of presentation to the ED = 21.9 ×10^3^/µL. - Received antibiotics (Cefepime, Vancomycin) and fluids in the ED. - ED diagnosis: anemia (unspecified type), sepsis due to unspecified organism. - Admitted to floor/non-critical unit. - Blood culture negative (x1); Joint/synovial fluid culture negative; Urine culture negative; Stool testing positive for Clostridium difficile. - Hospital length of stay = *15 days* (inclusive of day of ED presentation and day of discharge), then discharged home. - Discharge diagnosis: C diff colitis, right knee septic arthritis, pleural effusion. - Retrospective adjudication: Infection of Gastrointestinal / Abdominal and Skin/Skin Structure source; RESP. & RENAL organ dysfunction; **sepsis**. | 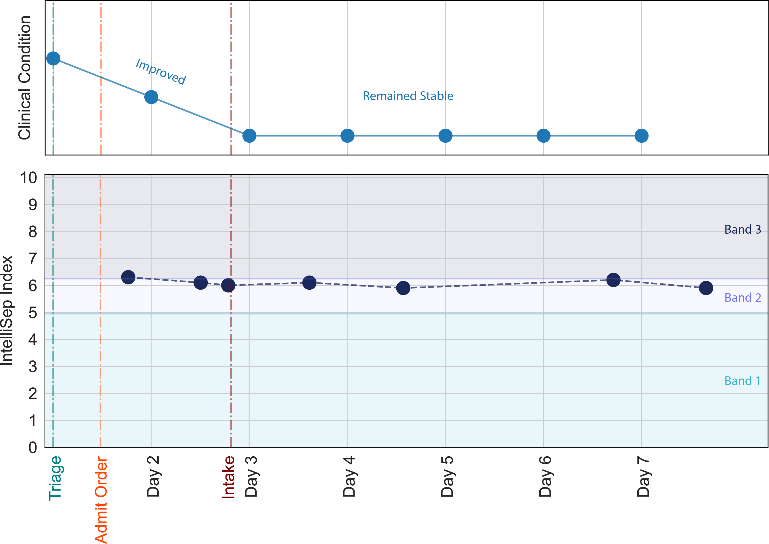**Sample for Day 5 was not drawn.*   \|  \| **Day 1** \| **Day 2** \| **Day 3** \| **Day 4** \| **Day 5** \| **Day 6** \| **Day 7** \| \| --- \| --- \| --- \| --- \| --- \| --- \| --- \| --- \| \| SOFA \| 1 \| 1 \| 2 \| 2 \| 0 \| 1 \| 0 \| |
|  | Patient ID 036 | - 49-year-old female - History of Anemia, HIV, Epilepsy. - Lactate measured in the ED = 0.8 mmol/L. - WBC on day of presentation to the ED = 4.6 ×10^3^/µL. - Received antibiotics (Cefepime, Metronidazole) in the ED. - ED diagnosis: fever of unknown origin with HIV infection. - Admitted to floor/non-critical unit*, elevated to ICU within 24 hours.* - Blood culture negative (x1); Urine cultures negative (x2). - Hospital length of stay = *15 days (4 ICU days)* (inclusive of day of ED presentation and day of discharge), then discharged home. - Discharge diagnosis: sepsis, HIV, pneumocystis, acute respiratory failure with hypoxia, cytomegaloviral disease. - Retrospective adjudication: Infection of Respiratory source; RESP. organ dysfunction; **sepsis**. | 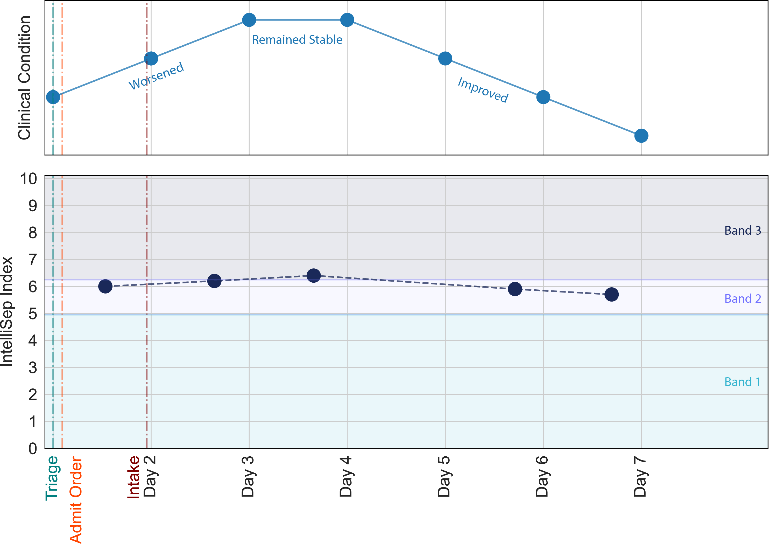  **Samples for Day 4 & 7 were not drawn.*   \|  \| **Day 1** \| **Day 2** \| **Day 3** \| **Day 4** \| **Day 5** \| **Day 6** \| **Day 7** \| \| --- \| --- \| --- \| --- \| --- \| --- \| --- \| --- \| \| SOFA \| 0 \| 2 \| 8 \| 5 \| 5 \| 5 \| 5 \| |
|  | Patient ID 038 | - 35-year-old male - History of Cystic Fibrosis. - Lactate was not measured in the ED. - WBC not measured on day of presentation to the ED. - Received antibiotics (Ciprofloxacin) in the ED. - ED diagnosis: pneumonia of left lower lobe due to infectious organism, cystic fibrosis, pancreatic insufficiency. - Admitted to floor/non-critical unit*, elevated to ICU after 12 days*. - Blood cultures negative (x1); Sputum cultures (1) negative, (2) positive for Pseudomonas spp. & Staph. Aureus; Respiratory panel negative. - Hospital length of stay = *19 days (5 ICU days)* (inclusive of day of ED presentation and day of discharge), then discharged home. - Discharge diagnosis: cystic fibrosis with pulmonary manifestations, abscess of lung with pneumonia, pneumonia due to Pseudomonas. - Retrospective adjudication: Infection of Respiratory source; GI organ dysfunction (not caused by infection); **no sepsis**. | 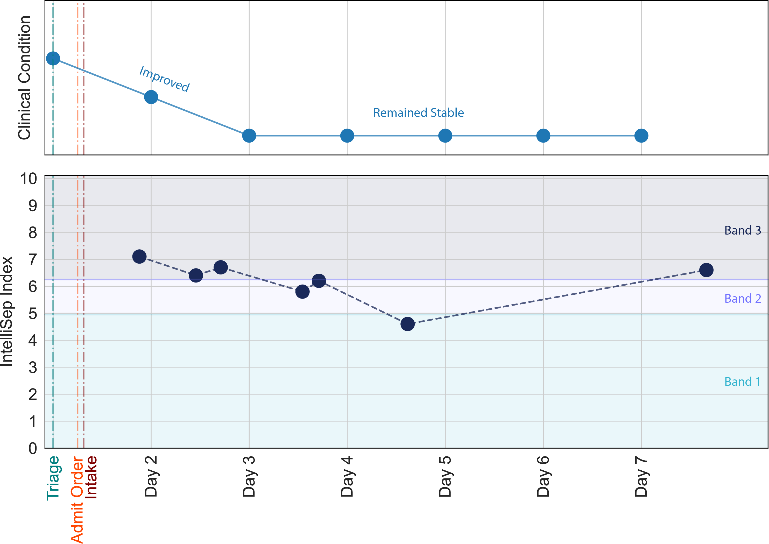**Samples for Day 5 & 6 were not drawn.*   \|  \| **Day 1** \| **Day 2** \| **Day 3** \| **Day 4** \| **Day 5** \| **Day 6** \| **Day 7** \| \| --- \| --- \| --- \| --- \| --- \| --- \| --- \| --- \| \| SOFA \| 1 \| 1 \| 1 \| 1 \| 1 \| 2 \| 2 \| |

**Note:** This table summarizes clinical vignettes, retrospective daily assessments of clinical condition, and ISI values measured across 7 timepoints for patients with LOS ≥ 15 days. The length of stay exceeds 2x the standard data collection period during the study.


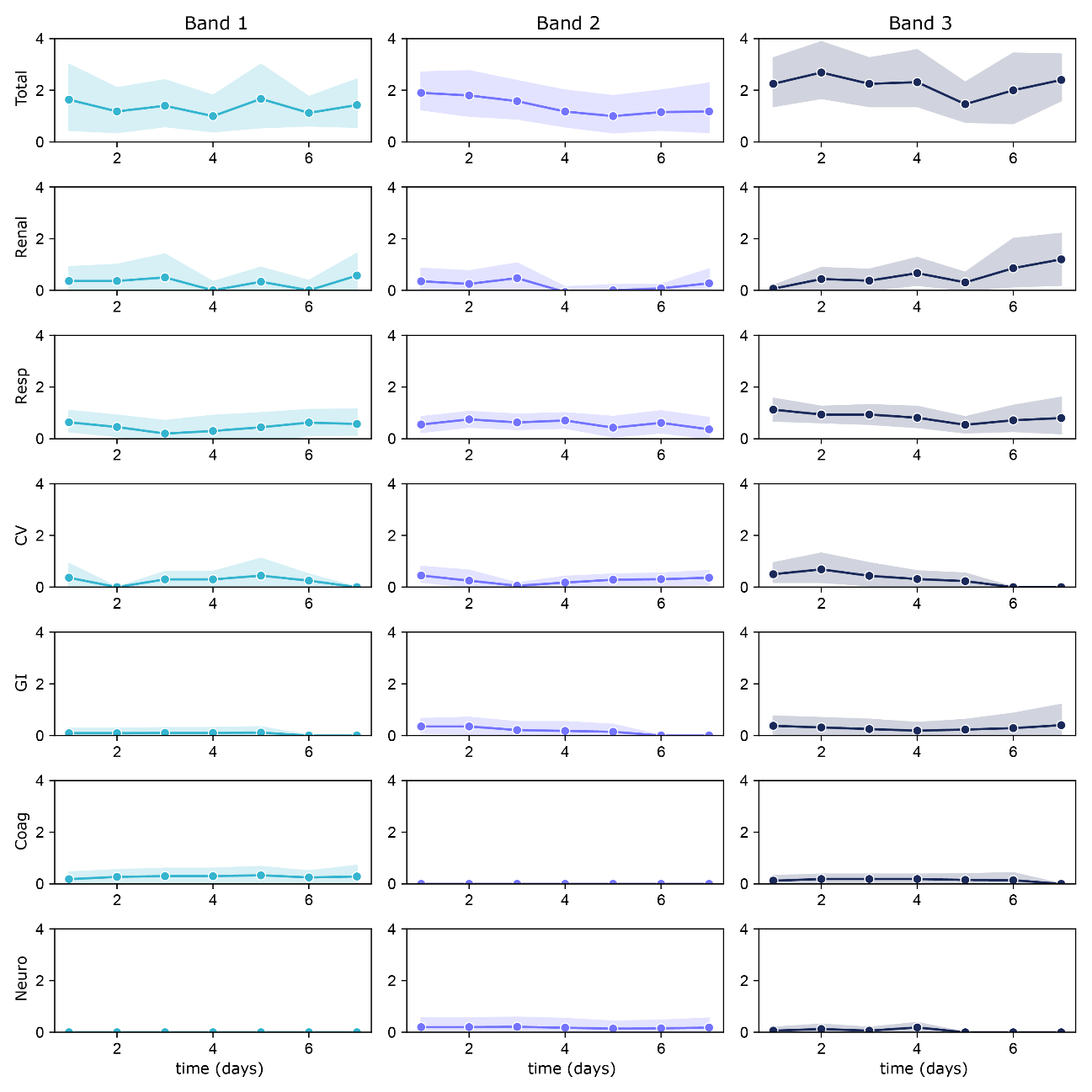


**Supplementary Figure S2.** Sequential Organ Failure Assessment (SOFA) scores over time, organized by the initial interpretation band. Organ systems are listed in descending order based on their contribution to the total SOFA score for patients initially classified in Band 3. Renal and respiratory systems were the primary contributors to the total score, with both systems showing a similar magnitude of effect. While we have observed that patients in Bands 2 and 3 show clinical improvement over the 7-day period, this improvement does not appear to be reflected in the SOFA scores, even when analyzed at the per-system level.


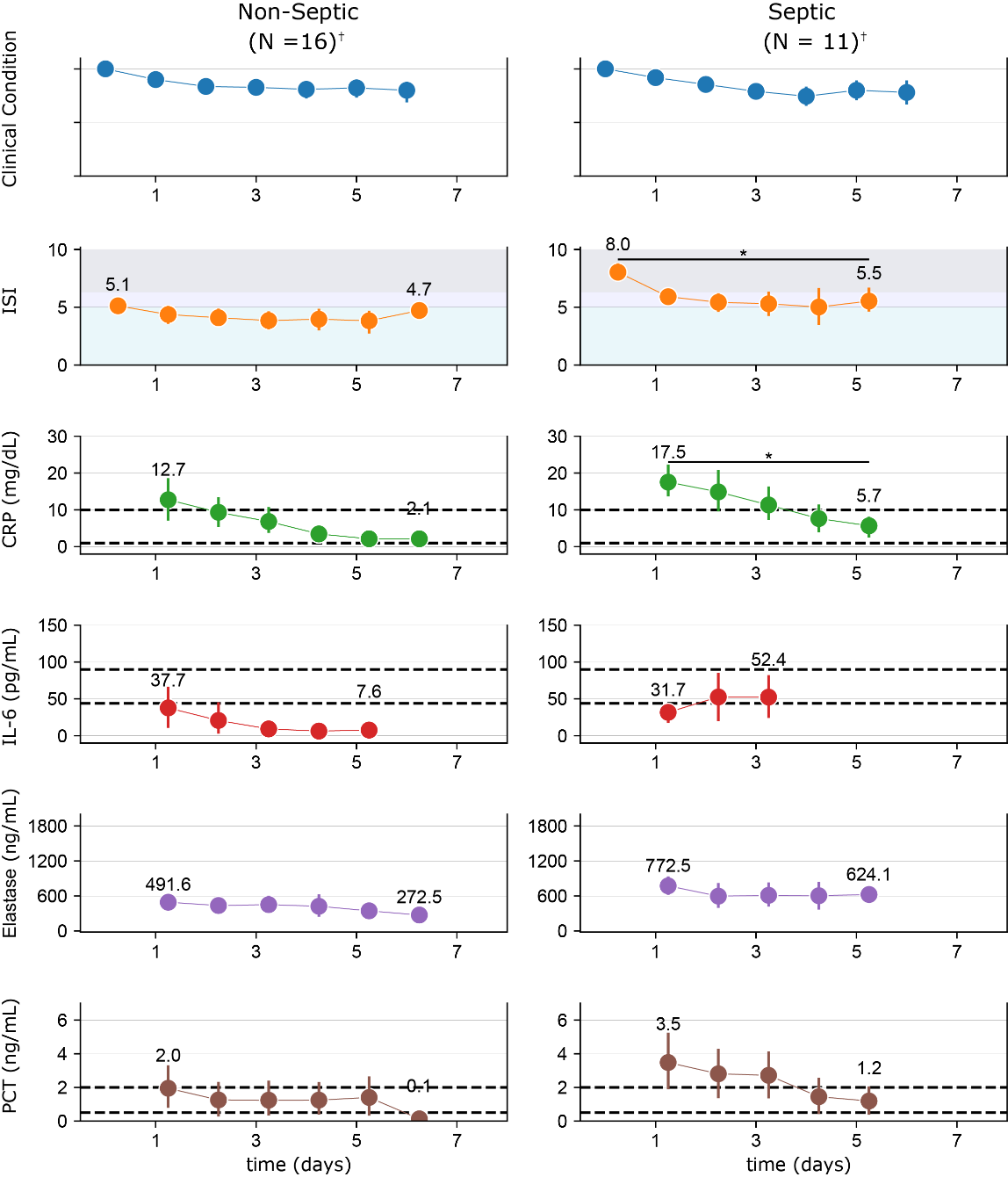


**Supplementary Figure S3.** Biomarker trends across time among septic and non-septic patients for ISI, C-Reactive Protein, Interleukin-6 (IL-6), Neutrophil Elastase, and procalcitonin (PCT), * indicates *P* < 0.05. †: N = 3 patients with indeterminate sepsis status were within this cohort. Unfilled time points among biomarkers are due to missing data.


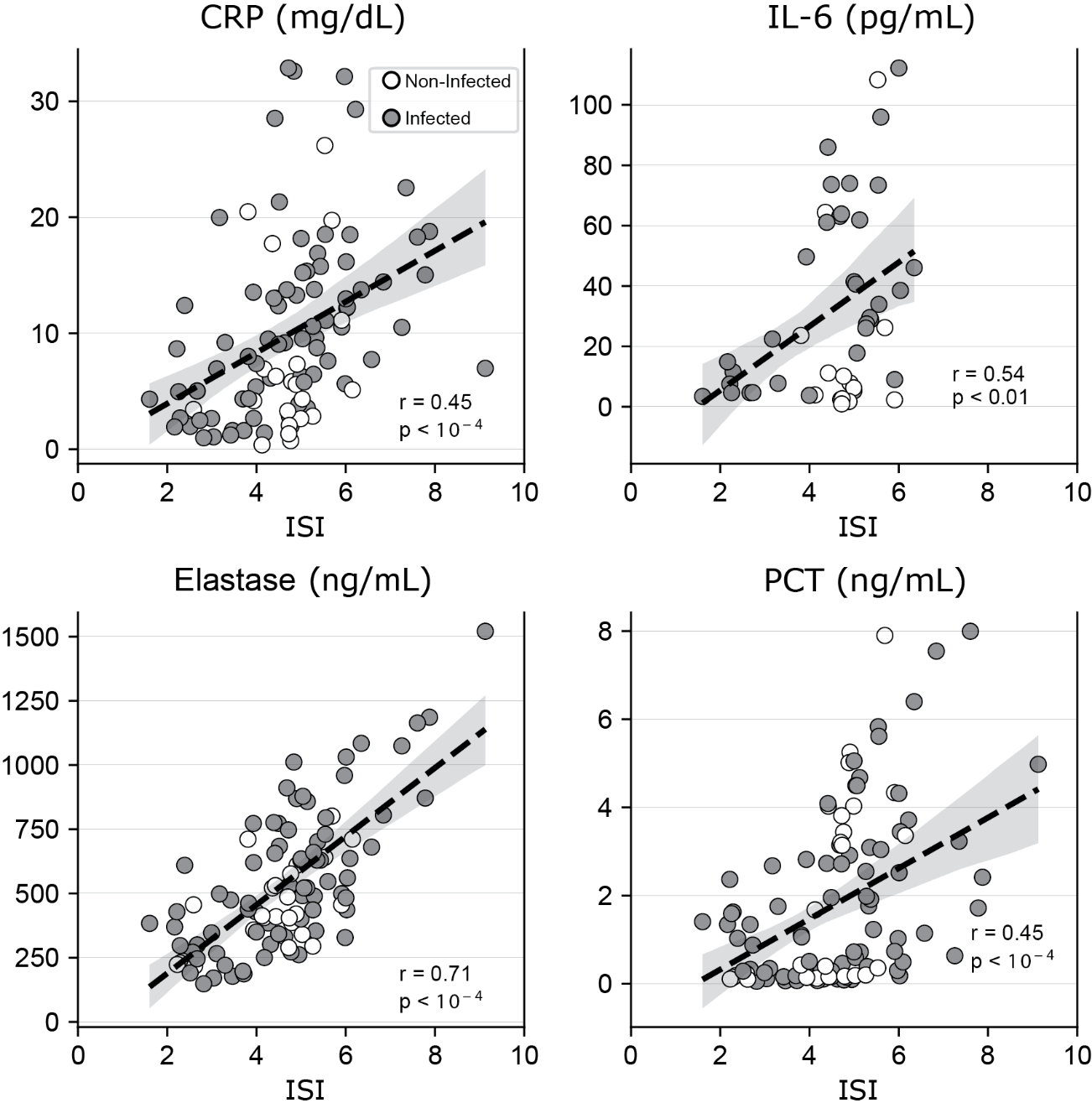


**Supplementary Figure S4.** Correlation between IntelliSep Index (ISI), C-Reactive Protein (CRP), Interleukin-6 (IL-6), Neutrophil Elastase, and Procalcitonin (PCT). “r” values indicate Pearson correlation coefficients. Gray and white markers indicate Infected and Non-infected patients, respectively.
